# Focused ultrasound treatment of methicilin resistant Staphylococcus aureus induced abscesses: pre-clinical study

**DOI:** 10.1186/2050-5736-3-S1-P58

**Published:** 2015-06-30

**Authors:** Laura Curiel, Charles Mougenot, Birgit Rieck, Kunyan Zhang, David Bates, Samuel Pichardo

**Affiliations:** 1 Thunder Bay Regional Research Institute, Thunder Bay, Canada; 2 Philips Healthcare Canada, Toronto, Canada; 3 University of Calgary, Calgary, Canada

## Background/introduction

Methicillin-resistant *Staphylococcus aureus* (MRSA) is a major nosocomial pathogen that particularly threatens immunocompromised patients who are prone to develop infections that are less and less responsive to regular treatments. Moreover, MRSA can cause abscesses that are difficult to treat. Because of its capability to induce a rise of temperature at a very precise location and the known sensitivity to heat of bacteria, FUS can allow for a localized treatment for MRSA-induced abscesses.

## Methods

MRSA abscesses (strain USA400) were induced after injecting a bacteria suspension at a concentration of 1.32±0.5x105 colony forming units (cfu)/mL subcutaneously in the left flank of BALB/c mice. An abscess of 6±2 mm in diameter formed after 48hrs. A small animal focused ultrasound system was then used to perform exposures on the abscess using a transducer operating at 3 MHz with a focal length of 50mm and diameter of 32mm. The focal point was positioned 2mm under the skin at the abscess center and four ultrasound exposures of 9s each were applied to each abscess under Magnetic Resonance Imaging guidance. Real-time estimation of change of temperature was done using water-proton resonance frequency (PRF) and a communication toolbox (matMRI) developed in-house. Three experimental groups of animals were tested: control, moderate temperature (52°C) and high temperature (64°C). The response to the treatment was assessed by culture and count of bacteria after treatment at two different time points: 1 and 4 days after treatment. Immune response after the treatment was evaluated by a Myeloperoxidase (MPO) assay that determined neutrophil recruitment as well as white blood cell count to evaluate the systemic inflammatory response.

## Results and conclusions

Treated abscess diminished on external size 1d after treatment and there were no open wounds. A significant reduction on bacterial count was obtained for the high temperature treatment and it was observed 4 days after the treatment. The median (lower to upper quartile) bacterial count 1 day after treatment was 6.18x103 (0.76x103 to 11.18x103), 2.86x103 (1.22x103 to 7.07x103) and 3.52x103 (1.18 x103 to 6.72 x103) cfu/100mL for control, moderate and high temperature groups, respectively; for the 4-day end point, the count was 1.37x103 (0.67x103 to 2.89x103), 1.35x103 (0.09x103 to 2.96 x103) and 0.07x103 (0.03x103 to 0.36x103) cfu/100mL for control, moderate and high temperature. The MPO amount and the white cell count remained unchanged between groups and days, indicating no change on local neutrophil recruitment and no systemic inflammatory response caused by the treatment. Focused ultrasound can induce a therapeutic effect in abscesses induced by MRSA. This effect is observed as a reduction of the number bacteria without significantly altering the amount of MPO at the site of an MRSA-induced abscess. These initial results suggest that focused ultrasound is a viable option for the treatment of localized MRSA-related infections.

**Figure 1:**
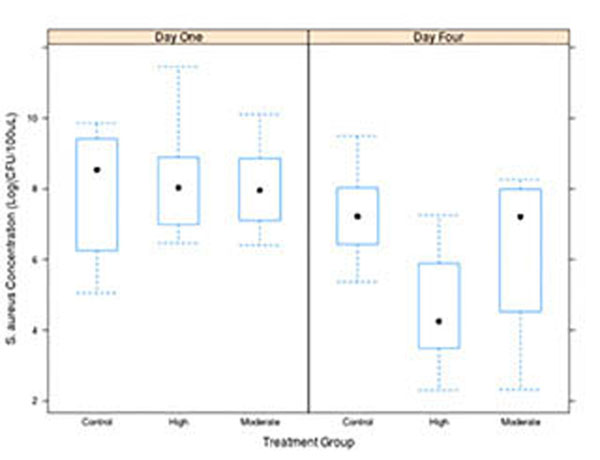
Bacterial count after exposure as a function of temperature and time point.

**Figure 2:**
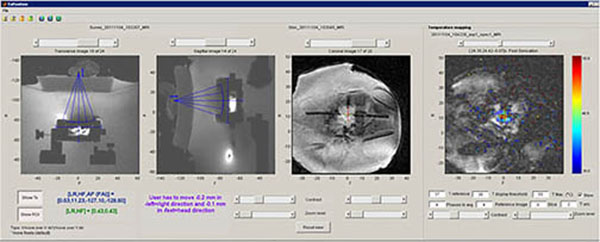
Monitoring of FUS exposure on MRSA abscess in mice.

